# Towards Standardization of Data Normalization Strategies to Improve Urinary Metabolomics Studies by GC×GC-TOFMS

**DOI:** 10.3390/metabo10090376

**Published:** 2020-09-19

**Authors:** Seo Lin Nam, A. Paulina de la Mata, Ryan P. Dias, James J Harynuk

**Affiliations:** Department of Chemistry, University of Alberta, Edmonton, AB T6G 2G2, Canada; seolin@ualberta.ca (S.L.N.); delamata@ualberta.ca (A.P.d.l.M.); dias1@ualberta.ca (R.P.D.)

**Keywords:** urine, metabolomics, normalization, mass spectrometry, GC×GC-TOFMS, creatinine

## Abstract

Urine is a popular biofluid for metabolomics studies due to its simple, non-invasive collection and its availability in large quantities, permitting frequent sampling, replicate analyses, and sample banking. The biggest disadvantage with using urine is that it exhibits significant variability in concentration and composition within an individual over relatively short periods of time (arising from various external factors and internal processes regulating the body’s water and solute content). In treating the data from urinary metabolomics studies, one must account for the natural variability of urine concentrations to avoid erroneous data interpretation. Amongst various proposed approaches to account for broadly varying urine sample concentrations, normalization to creatinine has been widely accepted and is most commonly used. MS total useful signal (MSTUS) is another normalization method that has been recently reported for mass spectrometry (MS)-based metabolomics studies. Herein, we explored total useful peak area (TUPA), a modification of MSTUS that is applicable to GC×GC-TOFMS (and data from other separations platforms), for sample normalization in urinary metabolomics studies. Performance of TUPA was compared to the two most common normalization approaches, creatinine adjustment and Total Peak Area (TPA) normalization. Each normalized dataset was evaluated using Principal Component Analysis (PCA). The results showed that TUPA outperformed alternative normalization methods to overcome urine concentration variability. Results also conclusively demonstrate the risks in normalizing data to creatinine.

## 1. Introduction

Metabolomics studies strive for comprehensive monitoring of metabolites in biological systems [1]. As common practice, metabolomics studies often involve the investigation of chemical differences between sample groups. One of the biggest challenges of group-based metabolomics studies is that the difference in metabolite concentrations between the comparative groups can be very subtle and detecting important differences is challenging [2,3]. Urine has been a popular matrix for metabolomics studies due to its non-invasive collection and availability in large volumes with a rich metabolite content. Due to these advantages, it has been reported as a precious clinical sample source for early non-invasive disease diagnosis [4,5,6,7]. Despite the convenience of urine as a diagnostic biofluid, there exists a major drawback as an analytical sample; urine concentration may vary significantly based on the level of hydration and may also be influenced by external factors such as diet, excretion via sweating, or kidney disease. This is in comparison to other biofluids, such as serum, plasma, and cerebrospinal fluids, which are physiologically and homeostatically managed by the body, resulting in much more controlled metabolite concentrations. When urine is sampled, factors such as the collection time and the flow rate can cause fluctuations in the concentrations of urinary metabolites [4,8,9,10]. It has been reported that the total concentration of urinary metabolites, regarded as urine sample concentration, can vary by more than 15-fold between samples [4]. Consequently, normalization is critical in urinary metabolomics studies to correct for different sample concentrations. Once the sample-to-sample variation of urine is considered and addressed adequately, then truly significant and relevant metabolomic changes of interest can be observed [11].

Various normalization strategies have been reported to account for fluctuations in urine concentrations [1,12]. The most common method is using creatinine concentration as a normalization factor [10,12,13]. Creatinine is a waste product of muscle metabolism; in the absence of kidney malfunction, the rate of urinary creatinine excretion is relatively constant between different individuals and within an individual over time [9,14,15,16,17]. Using creatinine as a normalization factor has been a gold standard for urinary metabolomics, with the underlying assumption that creatinine concentration reflects the urine sample concentration [10,18]. However, it has been reported that creatinine excretion is highly dependent on many factors, and thus it may not provide a reliable reference for sample normalization [4,8,9,10,14,19]. The variation of creatinine excretion in human subjects may occur due to differences in age, sex, ethnicity, level of physical activity, muscle mass, hydration, diet, diurnal rhythms, emotional stress, disease state, body mass, etc. Recent publications suggest that the use of creatinine as a single reference compound to normalize the wide concentration ranges of all urinary metabolites present in the sample is not advisable [8,9,14,20].

Another method uses osmolality, a measure of total urinary solute concentration, as a normalization factor [1,11]. The procedure of measuring osmolality, however, is not straightforward, and requires a separate analysis to measure freezing-point depression [12]. Another well-known approach for global urinary metabolic profiling is the constant sum normalization, which is to use the sum of all signals from metabolites detected in a sample as a normalization factor [21,22]. As a post-acquisition normalization technique, this carries a great advantage of not requiring any additional experimental procedures [9]. However, the major drawback of this approach is that the number of compounds detected in each sample can vary widely from one sample to the next. Therefore, using the total signal of all the metabolites as a normalization factor may not be an accurate reflection of the urine concentration. Thus, MS Total Useful Signal (MSTUS) as a normalization factor was proposed and is routinely used in multiple MS-based metabolomics studies coupled to liquid chromatography (LC) [8,23,24]. This method involves computational data processing to identify component mass spectral signals, associate related ions into molecular components, and then sum all peaks that are common to all samples to obtain the MSTUS normalization factor [8]. By incorporating only useful signals that are common to all samples, contributions from xenobiotics and artefacts can be diminished [4]. From several non-targeted metabolomic profiling studies, it has been reported that MSTUS normalization yielded better results when compared to other common normalization approaches [8,9]. The limiting factor of this approach is that it can only be applied to MS data and the method involves a proprietary algorithm to calculate the normalization factor [4,8,9].

Although the importance of sample normalization has been well-received and recognized by the metabolomics community, to date there is no standardized method for the normalization of urine concentration [9,25]. Unlike other omics work, sample normalization in metabolomics is much more complicated because of the greater diversity of chemical structures and concentration ranges. Nonetheless, normalization is an indispensable step in data pre-processing ahead of further advanced data analysis and mining to ensure that results are an accurate reflection of the relative concentrations of the samples so that true metabolomic variances of interest can be found. Failure to correct for such variation may lead to bias in the data that can lead to false discovery and misinterpretation of results [1]. Despite the awareness of the necessity of sample normalization, it has been ignored in many metabolomics studies due to the lack of a suitable, easy means of sample normalization. 

Herein, three sample concentration normalization methods are discussed, with a particular focus on urine. Urine samples from two different groups classified on the basis of sex were analyzed by two-dimensional gas chromatography coupled with time-of-flight mass spectrometry (GC × GC-TOFMS). The acquired data were normalized by the three different methods; 1) creatinine, a conventional method of urine normalization; 2) Total Peak Area (TPA), the sum of the areas of all detected peaks in a given sample, and 3) *Total Useful Peak Area* (TUPA), a strategy that the authors propose as an adaptation of MSTUS that makes it amenable to normalization of data from any chromatographic technique, regardless of the detector type (assuming that through a combination of detector selectivity and separation power of the chromatographic system, peaks can be unequivocally identified and tracked across the entire series of chromatograms). Contrary to MSTUS, TUPA does not mandate the use of an MS instrument, nor sophisticated computational data processing. While it is common in metabolomics studies to have MS detection, TUPA could equally be applied to normalize GC×GC-FID signals, LC-fluorescence, or any other chromatographic data. TUPA only requires a table of analyte identifiers (retention coordinates) and response data (peak areas) from a set of samples analyzed and processed with a consistent method. The performance of the three different normalization methods was then evaluated by a manual review of the data after normalization, along with principal component analysis (PCA). The aim of this paper is to examine the impact of normalization methods on the result of chemometric analysis and to propose a reliable sample normalization method for chromatographic data in metabolomics and, potentially, applications in other fields.

## 2. Results

Two samples, S15E and S26E, were removed due to an apparent instrument malfunction (frozen cold jet) in the data. PCA was used to visually inspect the clustering of QC samples. All QC samples were within the ordinary range of cluster, which is indicative of the absence of abnormal analytical variability. Aligned GC×GC-TOFMS data of 54 urine samples including replicates of one male and one female sample (S11MR, S17MR), were then normalized using the three different strategies: urinary creatinine concentration, TPA, and TUPA. The effects of different normalization strategies on the chemometric analysis were evaluated by visualizing the effects of normalization on the data in PCA score space both pre- and post-feature selection. 

The creatinine peak area, TPA and TUPA for each of the 54 urine samples are listed in Table 1. The minimum creatinine peak area out of all 54 samples was found to be 1.33 × 10^6^, whereas the maximum creatinine peak area was 22.1 × 10^6^ (a 16.6-fold difference). The average creatinine value of the 54 urine samples was 8.61 × 10^6^ with a relative standard deviation of 65%. Creatinine levels were compared based on the sex of the donor, but there were no statistically significant differences in this respect. For female samples, 2.07 × 10^6^ was the minimum creatinine peak area and the maximum creatinine peak area was 22.1 × 10^6^. The average creatinine peak area value of 30 female urine samples was 8.53 × 10^6^ with a relative standard deviation of 65%. For male samples, 1.33 × 10^6^ was the minimum creatinine peak area and the maximum creatinine peak area was 20.5 × 10^6^. The average creatinine peak area value of 24 male urine samples was 8.72 × 10^6^, with a relative standard deviation of 66%. 

Total Peak Area (TPA) and Total Useful Peak Area (TUPA) were calculated based on the aligned peak table. From the 54 urine chromatograms, 5572 peaks were aligned using the statistical compare feature in ChromaTOF^®^. For TPA, the areas of all aligned 5572 peaks were summed to generate a TPA normalization factor for each sample. Out of 54 samples, the minimum TPA was found to be 5.37 × 10^8^, while the maximum TPA was 3.61 × 10^9^. For calculation of TUPA, the sum of all peak areas for those peaks that are present in all samples under study was taken as the normalization factor. Out of 5572 aligned peaks, 470 peaks were found to be present in all 54 samples, covering about 8.4% of the total analyte response. The peak areas of these 470 compounds in each sample were summed, and the total peak area of these useful compounds was used as a normalizing factor for the respective sample. Since these compounds are common in all samples and the peak area was determined using the same mass channel for the same analyte in all samples, it was considered that the total peak area of these compounds could be regarded as a realistic representation of urine sample concentration. The smallest TUPA out of all 54 samples was 1.67 × 10^8^, whereas the largest TUPA was 8.48 × 10^8^.

**Figure 1:**
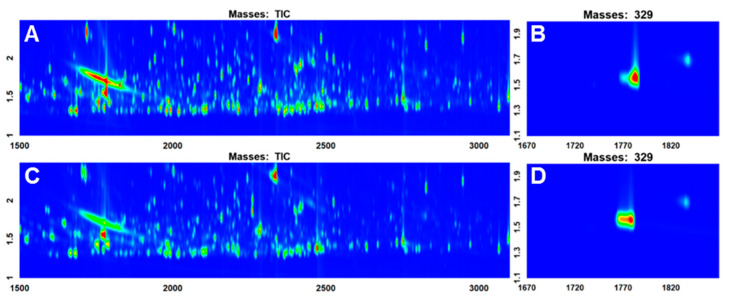
Comparison of two samples from the same subject. (**A**) S5M TIC (Total Ion Chromatogram), (**B**) S5M creatinine peak, (**C**) S5E TIC, (**D**) S5E creatinine peak.

Two urine samples (S5M and S5E) from the same individual at different concentrations were randomly selected for a more detailed and thorough comparison of the different normalization methods. The region of interest in the TIC chromatograms (1500 s–3100 s ^1^*t*_R_ and 1.0 s–2.5 s ^2^*t*_R_) and the zoomed-in regions for creatinine (plotted as ion 329 EIC) are illustrated in Figure 1. The color scales are constant for the TIC and EIC to facilitate visual comparison. From Figure 1, it is evident that S5M produced slightly more intense signals than S5E in general. The TPA were 2.39 × 10^9^ and 1.95 × 10^9^, and the TUPA were 7.46 × 10^8^ and 5.15 × 10^8^ for S5M and S5E, respectively. The peak areas of creatinine obtained from the creatinine-specific processing method were also compared. The creatinine peak area of S5M was found to be 1.33 × 10^6^ whereas the creatinine peak area was 16.49 × 10^6^ for S 5E.

To evaluate the impact of the different normalization strategies on the chemometric interpretation of the results, the three normalization methods were compared in PCA space in addition to the dataset without normalization. With all 5572 variables included, no distinct separation was possible for any of the normalizations. The PCA score plots of each dataset normalized with different methods are included in the Supplementary Materials, Figure S1. Feature selection was applied to each normalized version of the data to discover which variables were of value to separate the two sample groups while removing noise and irrelevant variables. This was achieved using an in-house algorithm for cluster-resolution guided feature selection (CR-FS). In applying the CR-FS algorithm, the 54 urine samples were randomly split into a training set for feature selection and model construction (36 samples—2/3 of the dataset), and a test set for model validation (18 samples—the remaining 1/3). Assignment into either set was random, and the process was permuted 100 times to diminish the possibility of overfitting or obtaining a good model by chance due to a particular division of samples into training, optimization, and validation sets. A survival rate of 50% was chosen for the feature selection algorithm (i.e., variables that were selected more than 50% of the time were included in the list of selected variables) Since the starting data matrices differ in terms of the values of the analyte responses depending on the normalization strategy (or lack of normalization), the number of variables selected for each data set was different, although the same automated feature selection algorithm was used. After feature selection, PCA was performed on the data to allow visualization of the effects of normalization on the data after feature selection. Without normalization, the optimal model required 223 variables; explaining 11.95% of total variance by PC 1 and 7.84% by PC 2. With normalization to creatinine, 168 variables were chosen, and 10.84% of the variance was explained by PC 1 and 7.34% was explained by PC 2. In neither case is there a clear distinction between the two groups in the first two components of PCA space. When normalized with TPA, 285 variables were selected and 13.89% was explained by PC1 and 7.05% was explained by PC2, with a slight overlap of 95% confidence level ellipse. When TUPA was used, 306 variables were selected which could clearly distinguish between male and female samples in PCA space.

**Figure 2:**
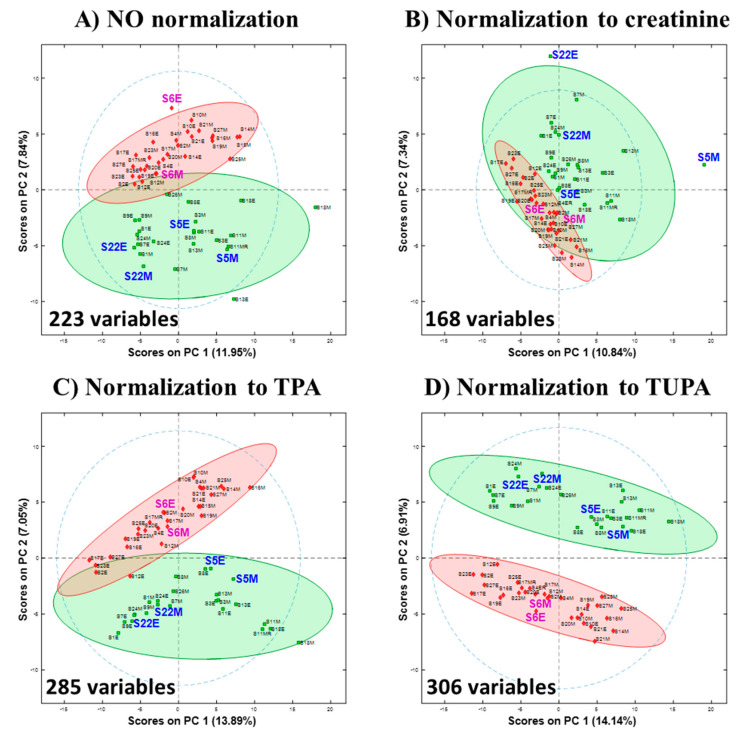
PCA score plots with 95% confidence ellipse for (**A**) without normalization, (**B**) normalized to creatinine concentration, (**C**) normalized to TPA, (**D**) normalization to TUPA.

Figure 2 also depicts the points for the pairs of samples from three subjects, labeled in a bigger font, to show the sample locations in the PCA score space. In the order of Figure 2A–D, the Euclidean distances between 5M and 5E are 18.4, 26.1, 13.0, and 18.1, respectively. The distances between 6M and 6E are 19.9, 13.1, 17.5, 12.2 and the distances between 22M and 22E are 8.3, 11.9, 7.1, and 6.6.

**Figure 3:**
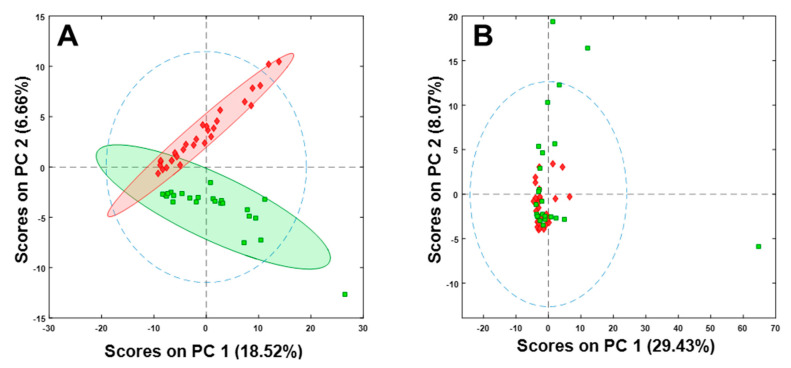
PCA score plots with 306 variables that were selected for TUPA modelled on the dataset (**A**) without normalization (**B**) normalization to creatinine.

To further demonstrate the need for proper normalization, the variables that were selected to model the data after TUPA normalization (Figure 2D) were used to model the system both without normalization, and with normalization to creatinine. (Figure 3). This was to demonstrate that the separation is not just based on the selected variables, but on the combination of proper normalization of analyte responses and the appropriate choices of variables. 

## 3. Discussion

In Table 1, the creatinine peak area, TPA and TUPA for each of the 54 urine samples are listed. Out of the three methods, the creatinine peak area showed the most variability. In order to use its peak area as a normalization factor for urinary metabolomics, integrating the creatinine peak area accurately is extremely important. In addition to the inaccurate biological assumption of constant creatinine excretion, it seems doubtful whether a single analyte can be used as a normalization factor over the entire range of metabolites. It is noteworthy that the total variation in TUPA is the smallest amongst the three different normalization methods at ~1/3 the variation in creatinine concentration. 

Two samples were randomly selected from the same individual at different concentrations (S5M and S5E) for a more thorough investigation of the effect of each normalization method (Figure 1). S5M is expected to be more concentrated than S5E because urine produced in the morning is generally more concentrated than urine produced throughout the day due to the overnight dehydration [26,27,28]. According to the TUPA, S5M is approximately 1.5 times more concentrated than S5E, which agrees with a visual inspection of the data, and is consistent with normal daily variation patterns in urine concentration. Conversely, the creatinine peak suggests that S5E is about 12 times more concentrated than S5M, which is obviously incorrect judging by visual inspection of the data.

PCA was used to evaluate the performance of each normalization method. PCA is an unsupervised method that helps visualizing and interpreting the data in exploratory data analysis. In this study, we used PCA for dimensionality reduction with the aim to project the data using the first two principal components. PCA as an unsupervised method was not used to classify male versus female, but PCA was employed to visualize similarity/dissimilarity between samples on the PCA score space [29]. 

The major challenge of urine analysis is the variability of urine concentration, which can depend on the time of sample collection, diet, exercise, and level of hydration, etc. [9,25,30]. Ideally, the differences between samples from the same subject at different times during the same day without any major change in diet, disease state, etc. should be relatively small with effective normalization. In PCA score space, one would expect that samples coming from the same subject should project relatively closer together with an optimal sample normalization method. To show how the normalization method would affect these results, the Euclidean distance between the projected pairs of points for three subjects was calculated for each PCA model. To reflect distances in the original data, 20 PCs were selected to build each model, explaining 71–74% of variance. It is noteworthy that for all four PCA score plots (Figure 2), approximately 20% of the variance was explained with the first two principal components. PCA score plots were set at the same scale, therefore the distances between samples can be easily measured directly from the plots, and similarities and differences among the samples can be quickly assessed [29]. It is observed that by using TUPA, samples from a single subject tend to project closer together in PCA space. It is also evident from the PCA score plots that TUPA displays tighter clusters (smaller within-group variation), especially for the male group, and a more distinct separation between the two classes (large between-group variation). This suggests that TUPA outperforms the other normalization strategies to correct for the urine sample concentration. 

Although the PCA score plot of the urine data with no normalization technique applied (Figure 2A) shows a moderate separation between the two groups, this result is analytically invalid because the variations in sample concentrations were not corrected. It has been extensively studied in the metabolomics community that urinary metabolite profiles vary widely based upon physiological and external factors such as diet, BMI, age, stress, exercise and etc. [14] For this particular study, it was intended to collect samples of widely varying concentrations from the donors of diverse backgrounds to obtain a dataset that contains sufficient diversity for investigating normalization approaches. With the collected widely varying samples for the study, neglecting normalization is irrational regardless of the performance of the resulted model. The PCA score plot that was normalized to creatinine (Figure 2B) shows the worst separation between the two groups out of all four PCA score plots. The normalization to creatinine made the separation worse than when no normalization was applied. This is likely due to creatinine normalization contributing nonsensical variations into the data (Figure 1). The use of creatinine for normalization was based on the assumption of constant creatinine excretion [10,13,17,18,19]. The poor performance of the creatinine-normalized model and the conclusion drawn from it that creatinine may not be a reliable normalization method is in agreement with the recent study that compared three normalizations—creatinine, specific gravity, and probabilistic quotient normalization (PQN) [31]. The TPA-normalized PCA score plot (Figure 2C) shows significantly improved separation between the two classes. However, this method would include all the insignificant peaks from the instrument and sample preparation in the normalization factor. The best separation was achieved by TUPA normalization. The whole dataset was normalized to a sum of only those peaks that are present in all samples; thus, the contributions from non-desired signals such as false peaks, noise, column bleed, and uncommon exogenous compounds could be eliminated. 

The present study adds weight to the current consensus in the metabolomics literature that failure to properly normalize the data and correct between-sample variation can create bias and result in the false discovery of biomarkers. A considerable variation in urine sample concentration, which can differ by up to an order of magnitude, must be managed by using a suitable normalization technique to minimize biases in the data. Of note, this study was conducted with a relatively small sample size and the question of the study may be viewed as too simple compared to more challenging and complex questions in metabolomics that involve very subtle differences between comparative groups. Yet, TUPA was the only normalization method that was able to produce data which could be separated into two distinct groups in this study. A thorough review of the data reveals TUPA to be a more reasonable method to account for urine sample concentration variability than using creatinine concentration. 

## 4. Materials and Methods 

### 4.1. Subjects and Sample Collection and Storage 

Two urine samples from each subject were collected for this study from a group of healthy volunteers (i.e., volunteers without any known preconditions). The instruction was given to each participant to collect the first urine of the day in the morning (M) to obtain highly concentrated urine, and another sample during the same evening (E) with enough hydration to obtain relatively less-concentrated urine from the same individual. A total of 24 urine samples from males and 30 urine samples from females were collected from the 12 male and 15 female participants for the study. The instructions for the study participants also included directives to store the sample approximately at 4 ℃ (in a refrigerator or in an ice bag without freezing the sample) upon collection and to bring the sample to the lab within at most 12 h. Upon receiving the sample, aliquots were taken to store the samples in smaller portions to avoid excessive freeze/thaw cycles followed by storage at −80 ℃ until sample preparation. A pooled QC sample was made by taking equal aliquots (500 µL) from each sample to check for analytical variability. All samples were analyzed in random order with adequate blanks, replicates and quality control (QC) samples. Informed consent was obtained from all individual participants included in the study. The study was approved by the University of Alberta Research Ethics Board (Study ID: Pro00071285). 

### 4.2. Chemicals, Reagents, and Solvents

Urease (Millipore Sigma, Oakville, ON, Canada) suspensions of approximately 40 mg per 250 µL of 18.2 MΩ deionized water (Elga PURELAB flex 3 system, VWR International, Edmonton, AB, Canada) were prepared on the day of derivatization. HPLC Grade Methanol (>99.9%) was purchased from Millipore-Sigma Canada, HPLC Grade Toluene (Millipore Sigma, Oakville, ON, Canada), was dried with anhydrous sodium sulfate (Millipore Sigma, Oakville, ON, Canada) prior to use. Methoxyamine hydrochloride (Millipore Sigma, Oakville, ON, Canada) was dissolved in HPLC Grade Pyridine (Millipore Sigma, Oakville, ON, Canada) to make a 20 mg/mL solution. Also, 1 mL ampoules of *N*-Methyl-*N*-trimethylsilyltrifluoroacetamide + 1% chlorotrimethylsilane (MSTFA + 1% TMCS) were purchased from Fisher Scientific, Ottawa, ON, Canada. A 100 µg/mL solution of 4-^13^C methylmalonic acid was prepared in deionized water to use as an internal standard. 

### 4.3. Sample Preparation 

All urine samples were prepared according to a modified protocol for global derivatization of urinary metabolites, which involves a typical two-step derivatization process of methoximation followed by subsequent trimethylsilylation [21,32]. The frozen urine samples were thawed on ice on the day of analysis, then vortexed for 1 min. Then, 40 µL of each urine sample was transferred into a 2-mL centrifuge tube. Also, 20 µL of internal standard was added along with 10 µL of urease suspension. Samples were vortexed for three minutes then incubated at 37 °C for 1 h. Next, 960 µL of methanol was added to each of the samples. Samples were vortexed again for 5 minutes, then centrifuged for 10 min at 10,000× *g* and 4 °C. Then, 500 µL of the supernatant was transferred to a 2-mL GC vial, and dried under a gentle stream of nitrogen at 50 °C until dry. Next, 50 µL of 20 mg/mL methoxyamine hydrochloride in pyridine was added to the dried residue and incubated at 60 °C for 2 h. Subsequently, 100 µL of MSTFA was added and incubated again at 60 °C for 1 h, and 100 µL of the derivatized metabolite extract was transferred to GC vials with 300-μL inserts for analysis by GC×GC-TOFMS. 

### 4.4. GC×GC-TOFMS Conditions

All GC×GC-TOFMS analyses were performed on a LECO Pegasus 4D system (LECO, St. Joseph, MI, USA) equipped with a four-jet dual-stage modulator. A 60 m × 0.25 mm; 0.25 μm df Rxi-5SilMS was used as the first-dimension column and 1.6 m × 0.25 mm; 0.25 μm df Rtx-200MS as the second-dimension column (Chromatographic Specialties, Brockville, ON, Canada). Two-dimensional chromatographic separations were performed with a constant flow rate of 2.0 mL/min using helium as the carrier gas and a modulation period of 2.5 s. A GERSTEL MPS Autosampler was used for automated injection of 1 μL aliquots of sample. The oven was initially held at 80 °C for 4 min and heated to 315 °C at a ramping rate of 3.5 °C/min. The final temperature was held for 10 min. The secondary oven and modulator temperature offset were constant at + 10 ℃ relative to the GC oven temperature and + 15 ℃ relative to the secondary oven temperature, respectively. Mass spectra were collected at an acquisition rate of 200 Hz over a mass range between 40 and 800 *m/z*. A relative voltage offset of 200 V was selected as the optimized detector voltage with an electron impact energy of −70 eV. The ion source temperature was 200 ℃ with a transfer line temperature of 250 ℃. The total analysis time for each run was 81.1 min. 

### 4.5. Data Processing and Analysis

Data were initially processed using ChromaTOF^®^ (v.4.72; LECO, St. Joseph, MI, USA). Each of the 54 urine samples was processed with two different data processing methods; one for general-purpose data processing and one for targeted integration of the creatinine peak. Omics data sets exhibit differences in analyte concentrations that span several orders of magnitude within a sample. The general data processing method was developed to detect as many compounds as possible while minimizing artefacts such as split peaks and spurious signals, while the creatinine method was optimized to specifically integrate only the creatinine peak. 

For the general data processing method, the baseline offset was set to 0.9 and the expected peak widths throughout the entire chromatographic run were set to 10 s for the first dimension and 0.15 s for the second dimension. The peak-finding threshold of S/N was set to 100:1 with the minimum S/N ratio for sub-peaks to be retained set at 10. A chromatographic region of 3530 s to 3640 s in the first dimension and 0.8 s to 1 s in the second dimension was excluded from data processing due to signal saturation from residual urease and siloxane column bleed. All chromatographic peaks were searched against the NIST-MS 2017 Libraries. 

Small retention time shifts are possible in GC×GC and common in metabolomics studies. The Statistical Compare feature of ChromaTOF^®^ aligned the peak tables across runs and ensured that the same ion was chosen to quantify a given peak throughout the data set. Tolerances for retention time shift were ± 5 modulation period (PM = 2.5 s) in the first dimension, and 0.2 s for the second dimension to account for the possible retention time shift across all samples. The minimum similarity for the mass spectral match to combine sub-peaks was set at 600 using all *m/z* values with abundances greater than 1 %. After aligning the peak tables with the set parameters, the Statistical Compare result was then exported as a .csv file for further processing, normalization, and chemometric analysis in MATLAB^®^ R2017a, Windows 64-bit version (The Mathworks Inc., Natick, MA, USA). 

### 4.6. Normalization to Creatinine

The data processing method for targeted creatinine integration was used to account for variable creatinine peak sizes/shapes arising from concentration differences in various samples. The processing method for creatinine limited the search region to the window of 1720 s to 1850 s in the first dimension with an expected peak width of 50 s and 0.5 s in the first and second dimensions, respectively. The m/z of 329, which is the molecular ion of creatinine-3TMS (derivatized creatinine with three trimethylsilyl groups), was used as a quantification mass. The rest of the parameters were the same as for the general data processing method. The creatinine peak data processing method was applied to all 54 chromatograms and the peak tables were aligned using the statistical compare feature in ChromaTOF^®^. The aligned peak table was then imported to MATLAB^®^ R2017a. Peak areas of all metabolites in each sample were then normalized to the creatinine response for that particular sample.

### 4.7. Normalization to TPA and TUPA

With the Statistical Compare feature in ChromaTOF^®^, the individual peak tables were aligned and ChromaTOF^®^ generated an aligned peak table containing basic statistics including average, standard deviation, minimum and maximum responses for each compound across the data set. The table also included a count column, which records the number of samples in which the corresponding peak appeared. For TPA normalization, the total peak area for each sample was calculated by summing the areas of all aligned peaks as the normalization factor. The TUPA normalization factor includes only peaks that were present in all 54 samples across the full dataset in the summation. All metabolites processed and aligned according to Section 4.5 were then normalized to the corresponding TPA and TUPA of the respective sample by dividing the original peak areas of metabolites by either TPA or TUPA normalization factors. 

### 4.8. Multivariate Analysis 

Further multivariate statistical analysis was performed using PLS Toolbox (R8.5.2; Eigenvector Research Inc., Wenatchee, WA, USA). The data were normalized using the three different normalization approaches for comparison as described in Section 4.6 and 4.7. Principal component analysis (PCA) was applied with cross-validation to evaluate general clustering of normalized data by two different methods. An in-house feature selection algorithm, Cluster Resolution Feature Selection (CR-FS), was used for variable selection. This approach is based on the evaluation of ranked variables via a sequential backward-elimination and forward-selection mechanism to maximize cluster resolution between comparative groups [33]. The feature selection was permuted over the data set 100× with the samples being randomly partitioned between training and optimization sets to avoid spurious results due to optimization with any given partitioning of the data. Complete information on this feature selection tool is beyond the scope of this paper and more detail regarding the approach can be found in other literature [33,34,35].

## 5. Conclusions

An appropriate normalization method is vital to prevent the biological information of the study from being masked by variation in urine sample concentration. This paper demonstrated conclusively that normalization to creatinine response can be misleading as the creatinine response (and thus normalization factor) can be at odds with changes in overall concentration that are self-evident upon manual inspection of the raw data. This result is in agreement with other recent literature. The present study demonstrated that normalization using TUPA appears to be a more suitable way to account for the variations of urine concentration when performing GC×GC-MS-based metabolomics studies as opposed to using creatinine concentration or TPA as a normalization factor. This post-acquisition normalization method is easy and convenient to apply without requiring any additional experiments. Our findings suggest that TUPA may be an effective and feasible alternative to normalize untargeted metabolomics data.

## Figures and Tables

**Table 1:** Comparison of creatinine (CT) peak areas, TPA, and TUPA of 54 urine samples.

Sample	Sex	CT	TPA	TUPA	Sample	Sex	CT	TPA	TUPA
S1M	M	8.42 × 10^6^	1.34 × 10^9^	3.27 × 10^8^	S14M	F	1.75 × 10^7^	2.41 × 10^9^	7.01 × 10^8^
S1E	M	3.57 × 10^6^	8.28 × 10^8^	2.48 × 10^8^	S14E	F	1.32 × 10^7^	1.83 × 10^9^	5.99 × 10^8^
S2M	F	3.83 × 10^6^	1.81 × 10^9^	5.02 × 10^8^	S15M	F	1.58 × 10^7^	2.79 × 10^9^	6.85 × 10^8^
S2E	F	2.14 × 10^6^	7.14 × 10^8^	2.53 × 10^8^	S16M	F	1.47 × 10^7^	2.16 × 10^9^	7.05 × 10^8^
S3M	M	1.28 × 10^7^	1.67 × 10^9^	4.31 × 10^8^	S16E	F	6.64 × 10^6^	1.19 × 10^9^	3.65 × 10^8^
S3E	M	8.77 × 10^6^	2.68 × 10^9^	6.48 × 10^8^	S17M	F	1.02 × 10^7^	1.75 × 10^9^	5.71 × 10^8^
S4M	F	1.46 × 10^7^	1.67 × 10^9^	5.41 × 10^8^	S17MR	F	5.47 × 10^6^	1.05 × 10^9^	3.35 × 10^8^
S4E	F	3.04 × 10^6^	1.38 × 10^9^	3.66 × 10^8^	S17E	F	2.31 × 10^6^	6.06 × 10^8^	2.48 × 10^8^
S5M *	M	1.33 × 10^6^	2.39 × 10^9^	7.46 × 10^8^	S18M	M	2.05 × 10^7^	2.71 × 10^9^	8.30 × 10^8^
S5E *	M	1.65 × 10^7^	1.95 × 10^9^	5.15 × 10^8^	S18E	M	1.65 × 10^7^	1.86 × 10^9^	6.28 × 10^8^
S6M	F	5.74 × 10^6^	1.70 × 10^9^	8.48 × 10^8^	S19M	F	2.21 × 10^7^	2.28 × 10^9^	6.91 × 10^8^
S6E	F	1.19 × 10^7^	1.53 × 10^9^	4.71 × 10^8^	S19E	F	2.07 × 10^6^	5.37 × 10^8^	1.67 × 10^8^
S7M	M	3.76 × 10^6^	1.78 × 10^9^	4.61 × 10^8^	S20M	F	3.60 × 10^6^	1.26 × 10^9^	3.54 × 10^8^
S7E	M	4.06 × 10^6^	9.66 × 10^8^	3.33 × 10^8^	S20E	F	4.71 × 10^6^	8.62 × 10^8^	2.78 × 10^8^
S8M	M	4.59 × 10^6^	3.61 × 10^9^	4.31 × 10^8^	S21M	F	4.38 × 10^6^	1.80 × 10^9^	4.79 × 10^8^
S8E	M	1.48 × 10^7^	1.55 × 10^9^	4.20 × 10^8^	S21E	F	7.29 × 10^6^	1.42 × 10^9^	4.55 × 10^8^
S9M	M	7.16 × 10^6^	9.16 × 10^8^	2.70 × 10^8^	S22M	M	6.00 × 10^6^	1.22 × 10^9^	3.40 × 10^8^
S9E	M	3.61 × 10^6^	6.77 × 10^8^	2.19 × 10^8^	S22E	M	1.72 × 10^6^	8.50 × 10^8^	2.35 × 10^8^
S10M	F	1.36 × 10^7^	1.67 × 10^9^	4.47 × 10^8^	S23M	F	7.29 × 10^6^	9.50 × 10^8^	2.57 × 10^8^
S10E	F	1.38 × 10^7^	1.71 × 10^9^	3.88 × 10^8^	S23E	F	2.42 × 10^6^	5.38 × 10^8^	1.80 × 10^8^
S11M	M	1.35 × 10^7^	1.88 × 10^9^	5.96 × 10^8^	S24M	M	2.75 × 10^6^	8.70 × 10^8^	2.43 × 10^8^
S11MR	M	1.32 × 10^7^	1.87 × 10^9^	5.49 × 10^8^	S24E	M	9.66 × 10^6^	1.39 × 10^9^	3.82 × 10^8^
S11E	M	1.17 × 10^7^	1.64 × 10^9^	4.44 × 10^8^	S25M	F	1.51 × 10^7^	3.01 × 10^9^	7.31 × 10^8^
S12M	F	7.71 × 10^6^	1.46 × 10^9^	3.87 × 10^8^	S25E	F	5.27 × 10^6^	1.04 × 10^9^	2.77 × 10^8^
S12E	F	4.68 × 10^6^	1.04 × 10^9^	3.25 × 10^8^	S26M	M	4.26 × 10^6^	1.29 × 10^9^	3.45 × 10^8^
S13M	M	2.72 × 10^6^	1.54 × 10^9^	4.25 × 10^8^	S27M	F	1.17 × 10^7^	2.18 × 10^9^	6.17 × 10^8^
S13E	M	1.74 × 10^7^	2.07 × 10^9^	6.09 × 10^8^	S27E	F	3.16 × 10^6^	6.40 × 10^8^	2.21 × 10^8^

* denotes the samples selected for further detailed comparison. “R” denotes replicate samples.

## Supplementary Materials

The following are available online at https://www.mdpi.com/2218-1989/10/9/376/s1, Figure S1: PCA score plots for different normalization methods before applying any feature selection. Each dataset was 54 samples × 5572 compounds.
