# Supplementary material for: Towards Standardization of Data Normalization Strategies to Improve Urinary Metabolomics Studies by GC×GC-TOFMS
Source: Metabolites. 2020 Sep 19;10(9):376. doi: 10.3390/metabo10090376 (PMC7570207; doi:10.3390/metabo10090376)
Supplement: Supplementary file 1 [file metabolites-10-00376-s001.pdf]

Supporting Information

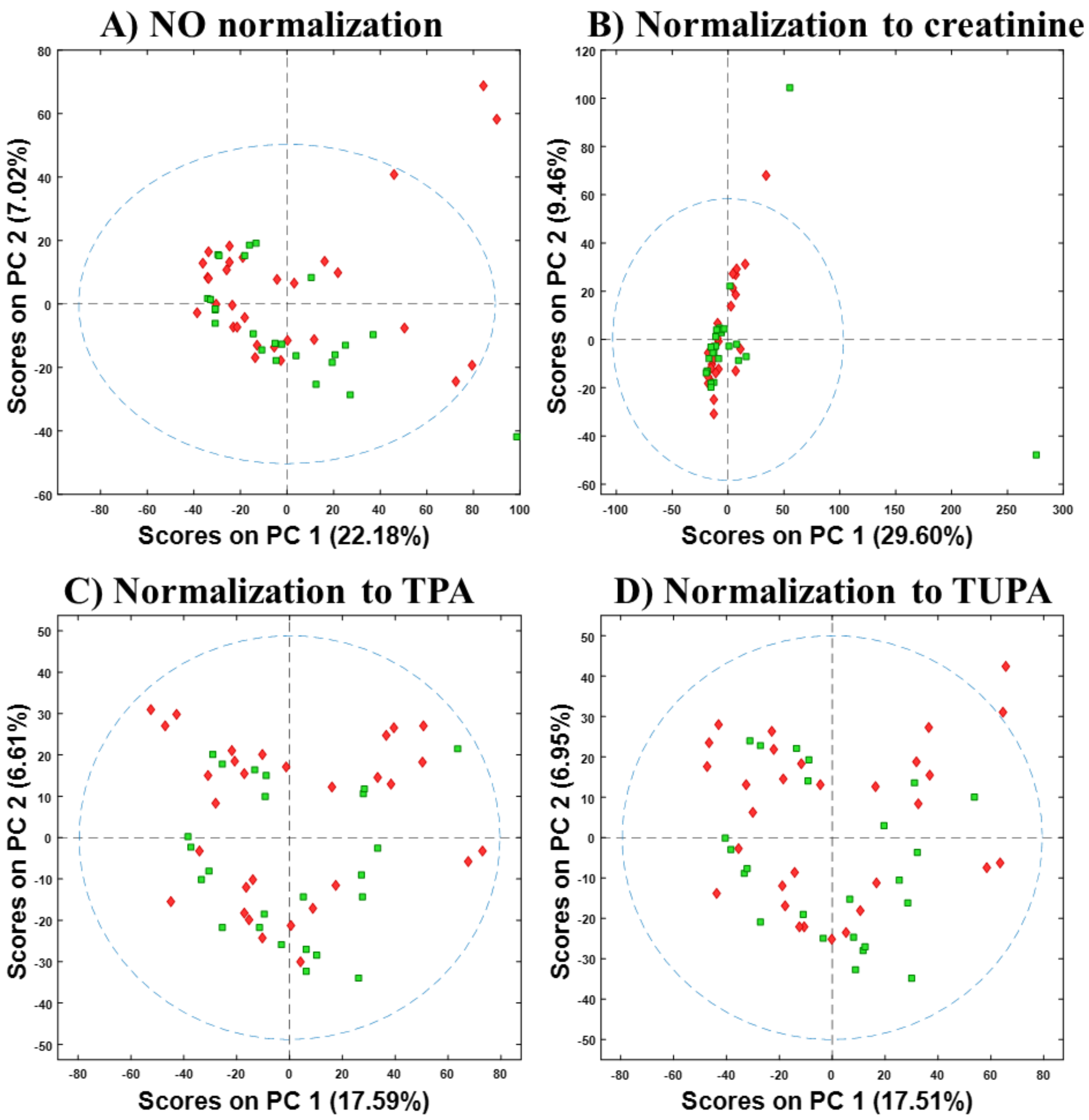

Figure S1: PCA score plots for different normalization methods before applying any feature selection. Each dataset was 54 samples × 5572 compounds.
